# A large-scale survey on finger counting routines, their temporal stability and flexibility in educated adults

**DOI:** 10.7717/peerj.5878

**Published:** 2018-10-31

**Authors:** Mateusz Hohol, Kinga Wołoszyn, Hans-Christoph Nuerk, Krzysztof Cipora

**Affiliations:** 1 Copernicus Center for Interdisciplinary Studies, Jagiellonian University, Cracow, Poland; 2 Section of Cognitive Science, Institute of Philosophy and Sociology, Polish Academy of Science, Warsaw, Poland; 3 Psychophysiology Laboratory, Institute of Psychology, Jagiellonian University, Cracow, Poland; 4 Department of Psychology, University of Tuebingen, Tuebingen, Germany; 5 LEAD Graduate School & Research Network, University of Tuebingen, Tuebingen, Germany; 6 Leibnitz-Institut für Wissenmedien, Tuebingen, Germany

**Keywords:** Number processing, Numerical cognition, Finger counting, Embodied cognition, Situated cognition, Handedness

## Abstract

A strong link between bodily activity and number processing has been established in recent years. Although numerous observations indicate that adults use finger counting (FC) in various contexts of everyday life for different purposes, existing knowledge of FC routines and their use is still limited. In particular, it remains unknown how stable the (default) FC habits are over time and how flexible they can be. To investigate these questions, 380 Polish participants completed a questionnaire on their FC routines, the stability of these routines, and the context of FC usage, preceded by the request to count on their fingers from 1 to 10. Next, the test–retest stability of FC habits was examined in 84 participants 2 months following the first session. To the best of our knowledge, such a study design has been adopted for the first time. The results indicate that default FC routines of the majority of participants (75%) are relatively stable over time. At the same time, FC routines can flexibly adapt according to the situation (e.g., when holding an object). As regards prevalence, almost all participants, in line with previous findings on Western individuals, declared starting from the closed palm and extending consecutive fingers. Furthermore, we observed relations between FC preferences and handedness (more left-handers start from the left hand) and that actual finger use is still widespread in healthy adults for a variety of activities (the most prevalent uses of FC are listing elements, presenting arguments and plans, and calendar calculations). In sum, the results show the practical relevance of FC in adulthood, the relative stability of preferences over time along with flexible adaptation to a current situation, as well as an association of FC routines with handedness. Taken together our results suggest that FC is the phenomenon, which is moderated or mediated by multiple embodied factors.

## Introduction

A large proportion of adults use finger counting (henceforth FC) in various contexts, such as calendar calculations, counting perceived objects, enumeration, or to communicate small numbers to other people (e.g., one usually shows two fingers at the same time as saying “two beers please” in a noisy pub; see Pika, Nicoladis & Marentette, 2009; Bender & Beller, 2011). This observation constitutes the starting point for systematic inquiries into the nature of FC. The human hand is the first computing machine for a number of reasons: our fingers are handy, perceptually distinguishable, and easy to move and manipulate. They can iconically represent discrete values or objects, their ordering is constant over time. A clear one-to-one correspondence between fingers and counted objects is easy to maintain (preserve), and hence, they are suitable for determining both cardinal number and ordinal number (Butterworth, 1999). FC is a spontaneous activity that has been present in the vast majority of cultures since prehistoric times (Göbel, Shaki & Fischer, 2011; Overmann, 2014). It had a great impact on the development of numerical systems and Western mathematics in its current form (Ifrah, 1981). Even today, FC appears to have a broader role than its normally ascribed function as a transitory step in the acquisition of numerical competences by individuals (Piaget, 1942; Gelman & Gallistel, 1986; Jordan et al., 2008). Hence, contemporary studies on FC are extensively carried out not only in children, but also in educated adults.

### Finger counting and numerical processing

There is a growing consensus that FC is not an immature strategy which serves only for assistance (Dupont-Boime & Thevenot, 2017), but affects number processing more directly (Previtali, Rinaldi & Girelli, 2011; Crollen & Noël, 2015; Newman, 2016; Newman & Soylu, 2014). It has been shown that FC reduces working memory load and provides control over the correctness of calculations (Wiese, 2004; Beller & Bender, 2011). Furthermore, the structure of finger-number relations affects basic symbolic number comparison (Domahs et al., 2010), where differences between different cultures could be traced back to different finger-number relations (see also Domahs et al., 2012). The direction of FC has also been claimed to affect spatial-numerical associations (or SNA; Fischer, 2008; Fischer & Brugger, 2011; Cipora, Patro & Nuerk, 2015; Lindemann, Alipour & Fischer, 2011) with the space-number relation (aka the mental number line) following the direction of FC.

What is more, finger-number relations seem to influence complex arithmetic in children and adults. Domahs, Krinzinger & Willmes (2008) showed that split-5 errors—namely, errors with a difference of plus-minus 5 from the correct result—are disproportionally frequent in children’s mental calculation and argued that this is due to underlying finger representations. Similarly, Klein et al. (2011) showed that not only carrying over unit sums beyond a base-10 quantity (e.g., 29 + 4), but also carrying over sub-base-5 thresholds (23 + 4) slows down responses in adults. Again, in the context of other sub-base-5 effects, they attributed these results to embodied finger number representations. What is more, there are several studies suggesting that early finger-related habits are positively related to later arithmetic skill (Willems, Feeters-Erenay & Depuydt-Bcrte, 1980; Fayol, Barrouillet & Marinthe, 1998; Noël, 2005; Jordan et al., 2008; Reeve & Humberstone, 2011; Chinello et al., 2013; Penner-Wilger & Anderson, 2013; Wasner et al., 2016; Fischer et al., 2017; Soylu, Lester & Newman, 2018). Because of this reason, some researchers suggested that FC may be considered as the missing link—in both ontogenetic and historical timescales—between the hardwired “number sense” (Dehaene, 2011) and culture-dependent, and more symbolical, numerical systems (Butterworth, 2005; Andres, Di Luca & Pesenti, 2008).

### Finger counting as a hallmark of embodied cognition

From a theoretical perspective, FC is often considered within the embodied cognition framework. Despite the fact that “the embodiment” is a label for different, and in some cases incoherent, approaches, most of its proponents agree that concepts—including abstract ones—are not arbitrary, amodal, and language-like symbols, as representatives of classic cognitive science typically assumed (Fodor, 1975; Jackendoff, 2002). Instead, they are to emerge from the bodily interactions of individuals with their environments (Clark, 1998; Barsalou, 1999, 2008; Wilson, 2002; Borghi et al., 2018). Although some amodal models of number processing have been proposed in the past (Groen & Parkman, 1972; Banks, Fujii & Kayra-Stuart, 1976), recently, there is growing agreement that mathematical concepts are indeed constrained by bodily activity and anchored, or systematically mapped, in sensorimotor systems (Lakoff & Núñez, 2000; Moeller et al., 2012; Landy, Allen & Zednik, 2014; Dackermann et al., 2017; Wołoszyn & Hohol, 2017; Fischer, 2012, 2018; Fischer & Shaki, 2018). The embodied approach to numerical cognition is supported, inter alia, by the results of neuroimaging studies which suggest that representations of fingers and numbers are shared. For instance, Zago et al. (2001) discovered that carrying out simple arithmetic operations involves activation of the same brain areas as those which are active during learning of finger movements sequences or during manual manipulation of three-dimensional objects. In line, Tschentscher et al. (2012) found that individuals’ habits regarding FC (specifically starting hand) is linked to neural activation during number processing. It has been suggested that simple counting is implemented through embodied, or off-line, simulation of finger movements (Andres, Seron & Olivier, 2007). There is also some evidence indicating that influences of FC routines on number processing go beyond simple arithmetic, and can also be observed in more elementary numerical processing (Fischer, 2008; Riello & Rusconi, 2011).

### Cultural embedding of finger counting habits

A comparison of Western and Middle Eastern adults reveals that the starting preference in FC is associated with the SNA direction. Specifically, Western two-hand counters start counting with their left hand, whereas Middle Eastern ones with their right hand (Lindemann, Alipour & Fischer, 2011). However, there is some evidence showing both within-individual variation in FC routines, as well as considerable variation within cultures, even in Western Europe, which share the same directions of SNAs. For instance, some left-to-right readers, such as French and Belgian participants, predominantly start counting with their right hand (Sato & Lalain, 2008; Lindemann, Alipour & Fischer, 2011). Several studies showed the same behavioral pattern in Italian adults (Di Luca et al., 2006; Di Luca & Pesenti, 2008, 2010; Sato et al., 2007; Fabbri, 2013; Fabbri & Natale, 2015; Fabbri & Guarini, 2016). On the other hand, the study by Newman & Soylu (2014) revealed no preference in starting habits among American right-handed adults. Therefore, even in countries that share a left-to-right reading/writing direction, FC habits differ between studies. The cause of observed diversity may, at least partially, lie in differences in FC assessment^1^ 1Note that there are cultural differences between left-to-right writing/reading countries even when the assessment is identical (Lindemann, Alipour & Fischer, 2011). These differences are not entirely surprising considering that there are multiple other mechanisms, which are possibly also responsible for left-to-right SNAs (Nuerk et al., 2015; Patro, Nuerk & Cress, 2016). Such non-reading/writing mechanisms have been shown to influence other SNAs (Patro et al., 2016), and their extent and prevalence might also differ between cultural contexts. However, since cultural differences are not the main topic of this article, a more detailed discussion goes beyond the scope of this introduction..

### Situatedness of finger counting habits

Besides embodiment, some evidence suggests that FC may also be prone to situational influences and that testing conditions largely affect FC routines used by participants. For instance, a series of experiments conducted by Lucidi & Thevenot (2014) showed that verbal reports of roughly a quarter of participants on FC routines were inconsistent with their actual pattern of behavior in the syllable-counting task. Wasner et al. (2014) also found that FC routines declared by participants might not always be consistent with their actual practices. As long as both hands were available, they spontaneously counted in line with their declared habits and only 28% of participants began counting with the left hand. When the task required counting with the fingers aligned horizontally (hands in front), 54% of tested persons began counting with the left hand. Finally, when participants were asked to count with the horizontal arrangement, but additionally with the dominant hand full, 62% of them started with the left hand. This was a between-participant design, but the results are significantly inconsistent with the idea that an individual maintains stable FC patterns regardless of the specific situated influences.

Following Wasner et al. (2014), it is essential, how to ask about FC routines. When participants view an image of their fingers in front of them aligned from left-to-right first, seems to influence the report of their FC routines. So, does having a pen in one hand. For these reasons, we asked all participants in our study first to count spontaneously (with two free hands) and memorize how they had counted and then to report their counting routines in the questionnaire. By this assessment, we were hoping to be closer to spontaneous counting procedures, that is, less influenced by horizontally aligned finger images than in previous group or internet studies.

### Stability of finger counting habits

The stability of FC routines over time is largely understudied. There is not much data, the existing findings are not conclusive (Previtali, Rinaldi & Girelli, 2011), and—to the best of our knowledge—not longitudinal. For instance, in a cross-sectional study by Sato & Lalain (2008) considering four age groups (4–5, 6–7, 10–11, and 24–47 years old) of French participants, the same pattern of FC was found regardless of age. Namely, participants were mostly starting with the right and continuing with the left hand. However, while this shows that the general pattern in a whole group does not significantly differ between age groups, the cross-sectional design reveals nothing about individual stability over time. Indeed, P. Räsänen & T. Koponen (2010, unpublished data; we quote after Previtali, Rinaldi & Girelli, 2011) found that although most Finnish right-handers, regardless of age, start counting from their right hand, such a pattern was not observed in left-handers. More precisely, although all left-handed preschoolers tested by Räsänen and Koponen were left-starters, only half of left-handed fourth graders were left-starters. Again, while these data suggest some group variability in development on a group level, little can be said about intraindividual stability. In summary, none of these studies tested the intraindividual temporal stability of FC routines since cross-sectional data have been collected only at the single time point. Therefore, the issue of temporal stability still needs to be addressed. This is the second goal of this study.

### Objectives of the present study

If FC routines play a role in the formation of directional SNA, as Fischer (2008) suggested, they should be stable, so that a given hand/finger occupying the particular relative position (e.g., third from the left) always corresponds to the same number. This would also imply that FC routines are stable within certain cultural contexts, which have been observed to share the same directions of SNAs (Shaki, Fischer & Petrusic, 2009). However, most previous reports on FC routines, as we have mentioned above, did not consider the temporal stability of FC routines. Furthermore, it should be noted that studies on FC are typically conducted only at a single time point.

First of all, we wished to provide quantitative data on FC routines with particular emphasis on their temporal stability, by directly (verbally) asking participants about their habits, and then testing it empirically in the retest study. Moreover, in the questionnaire we asked several questions on the stability of FC routines, which allowed us to cross-check the consistency of participants’ answers.

Secondly, Wasner et al. (2014) showed that left-to-right alignment of hands, while asking for FC routines, influences results. Therefore, we asked participants to count first without such an alignment, before they reported their FC routine. In that way, we attempted to make prevalence estimates obtained in this study more similar to spontaneous FC routines.

Finally, we wanted to provide data on how and when educated adults actually use FC in everyday life. The latter part of the survey was exploratory and aimed at providing quantitative data on typical FC routines, which would be useful in guiding future investigations.

## Method

### Participants

In the first session, there were 380 native Polish-speaking participants (236 female, 137 male; seven did not report their gender) aged 17–42 years (*M* = 20.8, SD = 3.6; 14 participants did not report their age) tested. The group consisted mostly of undergraduate students of law (*n* = 190) and psychology (*n* = 170); other participants (*n* = 16) studied math, computer science, cognitive science, or did not report their field of study (*n* = 4). Data from an additional five participants was excluded from further analysis due to the lack of data on FC direction, which was the main objective of the study. Two months following the first testing, participants, which we could reach again were invited to participate in the second session (psychology students, *n* = 84, 66 women, aged 19–42, *M* = 21.7, SD = 4.1).^2^ 2Due to organizational constraints (tight schedule at the end of the semester), we were not allowed to visit some lectures for the second time. The majority of the students, who were actually given the opportunity to participate in the second session agreed to do so. Part of the drop-outs may be simply related to students’ absences during classes or impossibility to match the pseudonyms from the first session (we did not have a code list, and some students could not recall the pseudonyms they had used in the first session). Thus, the much lower number of participants in the retest session was not due to self-selection. All participants gave informed consent verbally prior to the procedure. As the study was conducted in large groups of participants, we did not collect written consents to ensure anonymity. The design of the study was approved by the Local Ethics Committee of the Copernicus Center for Interdisciplinary Studies of the Jagiellonian University (decision no. 2 issued on the 30th of September 2015).

### Materials

FC routines were measured using a paper-and-pencil survey. The questionnaire consisted of the drawing of two palms taken from Fischer (2008), on which the participants were asked to mark their FC sequence, along with the set of questions described below (the original questionnaire in Polish, as well as its English translation, can be accessed at http://doi.org/10.17605/OSF.IO/RQHFK).

In the first question (henceforth Q1) participants were asked to indicate whether they (a) always follow the same sequence as they marked on the drawing, (b) usually do so, or (c) do not have any stable tendency in terms of the order of FC. In Q2 participants were asked to mark those out of six sentences, which described their FC routines (multiple answers were allowed). They were chosen based on an informal pilot procedure, in which several people naive to the purpose of the questions were asked how they count on their fingers and what they use FC for. Sentences were as follows: (a) I begin with a closed hand and extend the consecutive fingers; (b) I begin with a closed hand and extend the consecutive fingers touching fingers which have been already extended with the other hand; (c) I begin with an open hand and fold the consecutive fingers inwards; (d) I make gentle movement of consecutive fingers with a hand put on some object (e.g., a desk, a cup); (e) I make gentle movements of consecutive fingers keeping the hand open; (f) other way (specify). In Q3 participants were asked whether they would continue the FC sequence for numbers 6–10 by using the other hand (response alternative (a)), or by repeating the sequence with the starting hand (response alternative (b)). In Q4 participants were asked about their FC routine when the preferred hand is full. Three alternatives were presented: (a) I use the other hand without any problem; (b) I try to count on the preferred hand despite holding an object in it; (c) I put the object away or move it to the other hand. In Q5 participants were first asked to count on fingers of the hand opposite to the one they declared to begin with. Subsequently, they were asked how (a) natural; and (b) comfortable the counting was using a 5-point Likert-type scale with one representing very unnatural/uncomfortable and five—very natural/comfortable, respectively. The last question (Q6) concerned the circumstances in which participants use FC. Participants were asked to mark how often they use FC in each of the listed situations: (a) simple arithmetical operations, (b) calendar calculations, (c) listing elements (e.g., how many pairs of shoes/cousins do I have?), (d) describing plans/presenting arguments (e.g., First we will make repairs and next we will go for vacation), (e) multiplication, (f) communicating quantity, that is, finger montring (e.g., ordering three cups of coffee), (g) other (specify). Responses were given on a 5-point Likert-type scale from one (labelled with “never”) to five (labelled with “very often”).

The handedness was measured with the *Edinburgh Handedness Inventory* (Oldfield, 1971). The questionnaire consists of 10 questions in which participants declare their preferred hand in performing daily activities.

### Procedure

Data was collected during regular academic classes. Participants were tested in a group setup in lecture halls/seminar rooms. After providing general information about the study, participants, who agreed to volunteer, were orally instructed to have their hands free and keep their arms down (thus, they were not holding the hands in front of them and they did not see them), and then count on their fingers from 1 to 10 and memorize the order. Subsequently, the questionnaires were distributed, and participants were asked to mark the sequence on the palm drawing on the questionnaire (i.e., the schematic hands drawings were not visible to the participants while counting). Note that this procedure differs from Fischer (2008) in that participants first counted freely and then marked their preferred FC scheme. According to Wasner et al. (2014), this makes a meaningful difference (see Introduction). After marking the order of FC on the schematic hands, participants were asked to respond to items of the questionnaire. Participants were free not to return the questionnaires to ensure their freedom to withdraw from their participation in the study. They were also free to omit some items if they wished to. Thus, in some analyses the reported number of participants does not sum up to 380. The procedure lasted approximately 15 min.

## Results

### Handedness

The laterality quotient (LQ) could range from −100 to +100. According to Oldfield’s (1971) recommendations, the participants were categorized as right-handers (LQ > 40; *n* = 328, i.e., 86.3% of the sample), ambidextrous (40 ≤ LQ > 0; *n* = 22, i.e., 5.8%), or left-handers (LQ ≤ 0; *n* = 30, i.e., 7.9%)^3^ 3There is no consensus nor golden standard in the field (Lahita 1988; Tan 1991; Beratis et al., 2009; Arning et al., 2015). To check whether adopting alternative criteria actually would not change the results regarding handedness, we rerun the analyses using the following ranges: (A) LQ ≤ 0; 0 < LQ ≥ 50; LQ > 50 and (B) LQ < −40; −40 ≥ LQ ≥ 40; LQ > 40 for left-handers, ambidextrous, right-handers respectively. Disregarding the cut-offs adopted (A or B), the number of participants categorized as ambidextrous increased. Specifically, when we used the (A) criteria, 25 participants who—according to the initial cut-offs—were categorized as right-handed, now they felt into the ambidextrous group. When we used the (B) criteria, 10 out of 30 participants who were previously categorized as left-handers, felt into the ambidextrous category. These changes, however, did not change the major results of our study substantially. For instance, the relationship between handedness and starting hand was still significant (chi_2_^2^ = 12.62, *p* = 0.002; chi_2_^2^ = 12.63, *p* = 0.002; for A and B respectively). Moreover, all data, including the raw LQ values, are available at http://doi.org/10.17605/OSF.IO/RQHFK. So, every interested reader can conduct the analyses using handedness cut-off criteria she/he prefers to see whether adopting yet another handedness cut-off criterion makes a substantial difference for the handedness—FC relation..

### Finger counting routines

Results regarding the FC routines and patterns are summarized in Table 1. More participants declared starting with their right than with their left hand. Interestingly, the proportion of right- and left-starters differed significantly depending on handedness (chi_2_^2^ = 12.56; *p* = 0.002). This effect was driven by the difference between left-handers and two other groups. Left-handers were more likely to start FC from the left hand than from the right hand. The opposite was true for right-handers and ambidextrous (Table 2).

**Table 1:** Finger counting patterns and finger counting habits.

					*N*	%
**(a) Counting pattern**						
1	2	3	4	5		
RT	RI	RM	RR	RP	215	56.6
RT	RI	RM	RP	RR	3	0.8
LT	LI	LM	LR	LP	159	41.8
LT	LI	LM	LP	LR	2	0.5
LI	LM	LR	LP	LT	1	0.3
**(b) Starting hand**						
Right					218	57
Left					162	43
**(c) Starting finger**						
Thumb					371	97.6
Pinkie					8	2.1
Index					1	0.3
**(d) Hands used > 5**						
One					11	2.9
Both					369	97.1
**(e) Technique**						
Extending					288	75.8
Moving					21	5.5
Folding					5	1.3
Various					66	17.4

**Note:**

Counting patterns: each letter pair points at hand (R = right; L = left) and finger (T = thumb; I = index finger; M = middle finger; R = ring finger; P = pinkie) used to indicate the number at the top of its column. Data from items (a) to (d) are based on participants’ marks on the palm drawing. Data from item (e) are from the question.

**Table 2:** Handedness vs. starting hand.

Handedness	Starting hand				Total
	Right		Left		
	*N*	%	*N*	%	*N*
Right-handed	197	60	131	40	328
Ambidextrous	13	59	9	41	22
Left-handed	8	27	22	73	30

Irrespective of the starting hand, nearly all participants declared starting with their thumb. A few participants declared starting with their pinkie, and only one—with the index finger. Vast majority of participants used both hands to count to ten. Interestingly, when participants were explicitly asked whether they use the other hand or continue with the same one (i.e., Q3), four times more individuals declared using only one hand.

Out of 369 participants who declared using both hands, 353 declared they continued from the thumb of the other hand. Other participants declared starting from the pinkie of the other hand. Eight participants reported sequences not following the anatomical order in any direction.

The most prevalent FC technique reported by participants was extending their fingers. A total of 288 participants declared this as their only strategy. Out of these, 45 participants reported that they additionally touch the inner side of the other palm. The other techniques were far less common, with 21 participants declaring that they counted by making small movements either against a surface or against an item held in their hand. Only five participants declared that they start from an open palm and fold their fingers consecutively. Another five participants declared using “other” techniques only. The remaining 66 participants marked answers that were somehow contradictory with each other (e.g., both spreading and using an item). Most likely, these participants were using varied routines and did not reveal very strong preferences toward any of them.

**Figure 1:**
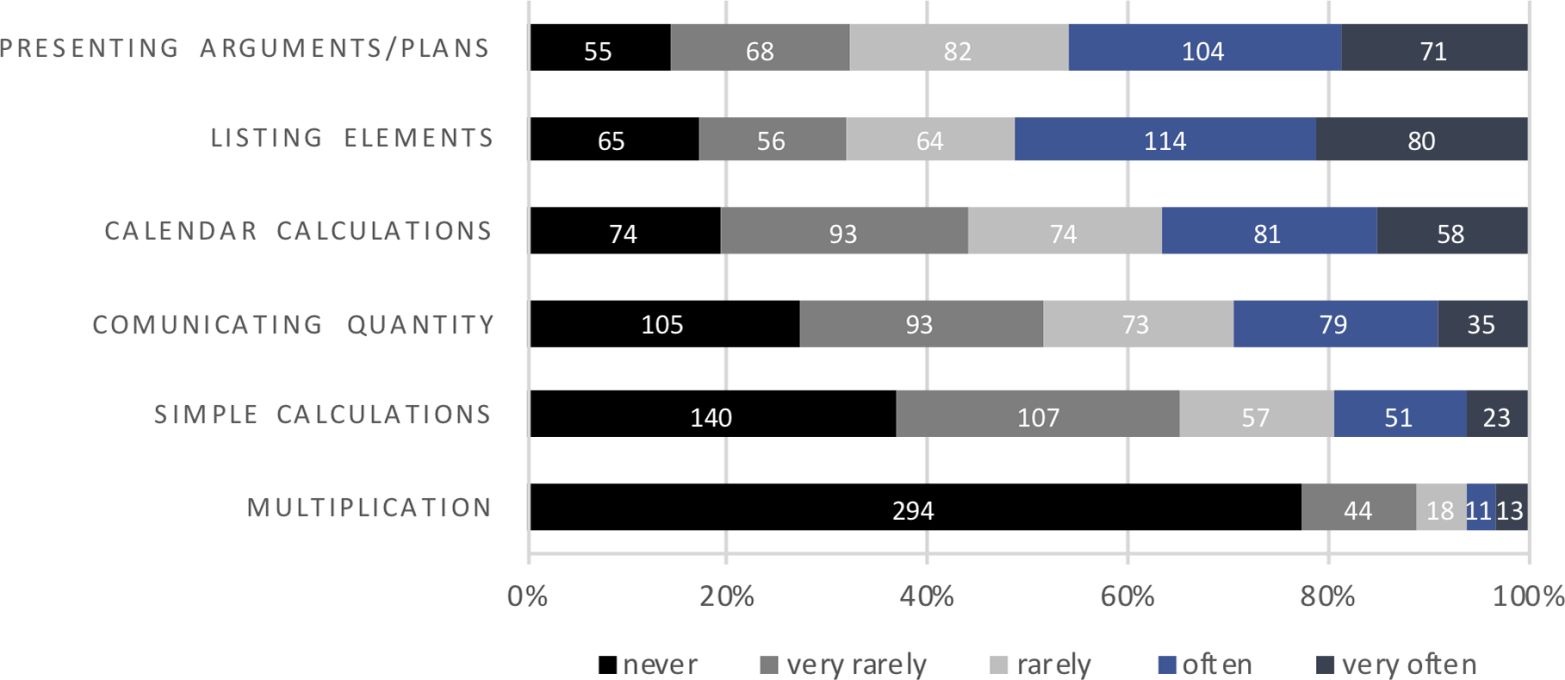
Circumstances in which finger counting is used.

The most common use of FC declared by participants was listing elements and presenting arguments and plans. A considerable proportion of participants used FC for calendar calculations. Communicating quantity and making simple calculations were somehow less prevalent. By far the least common use of FC reported by the participants was for multiplication (Fig. 1).

### Finger counting routine stability

Four questions referred to the declared stability of FC routines. Specifically, Q1 (explicitly asking about the stability), Q4 (actions taken when one needs to count on fingers, but the preferred hand is full), Q5 (a) and (b) (how natural/comfortable it was to start counting with the non-preferred hand; 5-point Likert-type scale). The majority of participants declared having a stable or partly stable FC routine (*n* = 186, i.e., 49% and *n* = 141, i.e., 37%, respectively) as opposed to not having such a routine (*n* = 52, i.e., 14%). Roughly half (*n* = 186, i.e., 49%) of the participants declared having no problem with using the non-preferred hand while having their preferred hand full, whereas the rest of them declared using the preferred hand anyway, or putting away or moving to the other hand the object being held in order to count on the preferred hand (*n* = 114, i.e., 30% and *n* = 79, i.e., 21%, respectively). Notably, participants’ responses to questions on how comfortable and natural it was to count with the non-preferred hand were rather high (i.e., participants were generally using the upper part of the scale). The mean response to the question how comfortable it was, was 3.5 (SD = 1.1) and to the question on how natural it was, was 3.69 (SD = 1.06).

Right- and left-starters did not differ in respect of any measure of declared FC routine stability (for Q1, chi_2_^2^ = 2.47, *p* = 0.291, for Q4, chi_2_^2^ = 0.77, *p* = 0.681, for Q5 (a), *t*_377_ = 1.03, *p* = 0.306 for Q5 (b), *t*_377_ = 0.41, *p* = 0.682).

### Consistency of the stability measures

The further analyses investigated the consistency between the four measures of stability. Congruency between Q1 (reported stability of FC routines) and Q4 (actions taken when preferred hand is full) was estimated by means of chi^2^ statistic. With increasing declared stability, the proportion of participants who claimed that they easily use the other hand systematically decreased, whereas the proportion of those who declared that they try to count anyway, or put away the object being held increased (chi_4_^2^ = 23.34, *p* < 0.001; Table 3).

**Table 3:** Reported finger counting routine stability vs. declared actions taken when one needs to count with fingers but the preferred starting hand is full.

Declared stability	Actions taken when preferred hand busy						Total
	Use other hand		Count anyway		Put away		
	*N*	%	*N*	%	*N*	%	*N*
Always	69	37	72	39	45	24	185
Usually	83	59	32	23	26	18	141
No preference	34	65	10	19	8	15	52

Congruencies between responses to Q1 (reported stability of FC routines) and Q5 (a) (how natural was FC with non-preferred hand) and Q5 (b) (how comfortable was counting with non-preferred hand) were estimated by means of Jonckheere–Terpstra (J–T) test for a monotone trend. This statistic tests whether means in the dependent variable change monotonically as a function of an independent variable. The declared stability was coded in increasing order. Higher declared stability was related to responses referring to feeling less natural and comfortable (observed J–T’s 16036.5 and 17371.0, respectively; *p’s* < 0.001; Table 4, upper part).

**Table 4:** Relations between finger counting stability measures.

Dependent variable	Group	*N*	Mean	SD
	Reported stability			
	No preference	52	4.70	0.91
Natural	Usually	141	3.74	0.98
	Always	185	3.48	1.07
	No preference	52	4.00	1.19
Comfortable	Usually	141	3.53	0.98
	Always	185	3.32	1.13
	Actions taken when preferred hand busy			
	Use other hand	185	4.04	0.92
Natural	Count anyway	114	3.54	1.07
	Put away	79	3.13	1.06
	Use other hand	185	3.72	1.10
Comfortable	Count anyway	114	3.36	1.10
	Put away	79	3.15	1.03

Relations between Q4 (actions taken when preferred hand is full) and Q5 (a) (how natural was FC with non-preferred hand) and Q5 (b) (how comfortable was counting with non-preferred hand) were investigated by means of a nonparametric Kruskal–Wallis (K–W’s) test. Individuals who declared no problems with using the non-preferred hand also marked that counting against their typical routine was more natural and comfortable (see Table 3, lower part). In both cases the differences were significant (K–W’s 44.38 and 20.68 for Q5 (a) and Q5 (b), respectively; *p* < 0.001).

The consistency of responses to Q5 (a) (how natural was FC with non-preferred hand) and Q5 (b) (how comfortable was counting with non-preferred hand), evaluated by means of polychoric correlation, was 0.66. This method is aimed to test correlations between discrete measures of continuous dimensions, like in Likert-like scales (Gadermann, Guhn & Zumbo, 2012).

### Temporal stability

Out of 84 participants for whom we obtained the data in the second measurement, 63 (i.e., 75%) were stable in their FC sequence. Note that on a group level, this is remarkably consistent with the self-report data. In self-report about 50% declared to be stable. The other 50% declared to be flexible. Thus, if they started counting randomly about half of this other flexible 50% (namely 25%) should start with the same hand and the other half with the different hand. Together, this corresponds to the 75% participants actually showing stability.

Interestingly, individuals who were right-starters in the first measurement were more stable than the left-starters (chi_1_^2^ = 7.04, *p* = 0.008). Notably, participants who declared that their FC routines are stable were, indeed, more stable over time (chi_2_^2^ = 7.95, *p* = 0.019; Table 5), supporting the validity of the verbal reports.

**Table 5:** Observed vs. declared finger counting routine stability.

Observed stability	Declared stability						Total
	Always		Usually		No preference		
	*N*	%	*N*	%	*N*	%	*N*
Stable	37	58.7	22	34.9	4	6.4	63
Unstable	6	28.5	10	47.7	5	23.8	21

We obtained the retest data only from four left-handed participants (three being stable and one unstable), and three ambidextrous (two being unstable and one stable) thus this analysis should be treated with caution. The Fisher’s exact test did not reveal any significant differences in stability related to handedness (*p* = 0.194 two-sided), but due to the low *n*, the power here is too low to draw reliable conclusions.

Participants whose FC was stable did not significantly differ from those whose FC was unstable regarding the description of their FC techniques (responses to question 2a-2e, *p* > 0.20). They also did not differ in terms of actions taken if they were to count with their fingers while their preferred hand was full (*p* = 0.317). The same is true regarding the responses to questions on how comfortable and natural it was to count with a non-preferred hand (Mann–Whitney’s-U 558.5 and 627.0 and *p* 0.312 and 0.794, respectively). Furthermore, these groups did not significantly differ in overall FC use index (see below; *p* = 0.505).

### Finger counting use and its correlates

We tested whether reported frequencies of FC use (questions 6a–6f) correlate with each other. Note that only 39 participants marked any answer to question 6g (other situations), for this reason we did not consider this question anymore. As responses were given on a Likert scale, polychoric correlations were used (cf. Table 6). Correlations were low to moderate, and sometimes even negative. It thus seemed that the FC use is heterogeneous and situation-dependent, and cannot be considered as an individual characteristic.

**Table 6:** Polychoric correlations between responses to items regarding for finger counting frequency in different contexts.

	1	2	3	4	5
1. Simple calculation	–				
2. Calendar calculation	0.41	–			
3. Listing	−0.23	0.51	–		
4. Arguments/plans	0.09	−0.23	−0.24	–	
5. Multiplication	0.48	0.30	−0.22	−0.21	–
6. Montring	0.05	0.13	−0.21	0.24	0.08

To check for potential differences in terms of FC use in each of the listed situations, K–W’s (for declared stability and handedness) and Mann–Whitney (for starting hand and gender) nonparametric tests were carried out for each item. The tests did not yield any significant differences for declared stability, handedness and starting hand. However, there were differences in the case of gender. Namely, females declared significantly more FC use than men in case of simple calculation (*p* = 0.023), calendar calculation (*p* = 0.005), listing (*p* < 0.001), and multiplication (*p* = 0.047). There were no differences as regards presenting plans and arguments (*p* = 0.492). On the other hand, men declared using more finger montring than women (*p* = 0.003).

## Discussion

### Overview

In a large-scale survey we aimed at investigating FC habits and their temporal stability among adult participants to provide relevant quantitative data, which—to the best of our knowledge—was still lacking in the existing literature. Replicating previous studies (Wasner et al., 2014), we observed that most of the Polish speaking students started FC from their right hand, when asked to count freely first and subsequently to report their FC preferences in the questionnaire. This proportion was modulated by handedness (starting with a left hand was more prevalent in left-handers). The most prevalent uses of FC were listing elements, presenting arguments/plans, and calendar calculations. Most participants declared in a questionnaire that their FC routines were stable, and we observed congruency in responses to different items asking about this consistency. However, here we also tested the stability of FC routines for the first time: Crucially, 75% participants of the retest session in fact repeated the same FC sequence when tested 2 months following their initial measurement. Furthermore, we provide some exploratory data on the uses of FC in everyday life situations.

### Starting hand and the finger counting direction

The majority of participants started FC sequences from the right hand. Even though the proportion of right- and left-starters differed significantly depending on handedness, there was still a lot of unexplained variance, which could not be accounted for by reading direction (Polish speakers as other Western cultures read and write from left to right). This result is similar to other reports showing a relatively large prevalence of right-starters among Western cultures (Di Luca et al., 2006; Wasner et al., 2015).

Here, the method of investigation is essential. When just presenting the FC questionnaire of Fischer (2008), the majority of participants (e.g., 54% in Wasner et al., 2015) reported starting with the left hand. However, in spontaneous counting, the vast majority (72% in Wasner et al., 2015) started with the right hand. In our study, about 57% started with the right hand. This is approximately in the middle between the 72% in spontaneous counting and the 46% in the questionnaire condition of Wasner et al. (2015) study. Two reasons might be responsible for that divergence: cultural and methodological differences. First, as shown by Lindemann, Alipour & Fischer (2011), there are considerable differences between cultures in their FC routines even if these cultures have the same reading direction. Secondly, we first asked the participants to count spontaneously and then to report it in the questionnaire. It remains possible that some participants were influenced by the questionnaire and reported left-to-right counting although they did not spontaneously do so. These reasons merit further investigation in future cross-cultural and cross-methodologicatol studies.

Finally, this observation provides further evidence that the role of FC routines for directional SNAs might not be as strong as previously assumed (Fischer, 2008). Although the majority counted right-to-left, typical left-to-right SNAs have been demonstrated several times in Polish speakers (Cipora & Nuerk, 2013; Cipora et al., 2016).

### Finger counting description

Nearly all participants declared starting counting with their thumb and using both hands while counting to 10. The revealed pattern of FC is consistent with previous findings on Western cultures (Wasner et al., 2014) and, at the same time, opposite to the routines of Middle-Eastern (e.g., Iranians, who typically counted from the pinkie to thumb; see Lindemann, Alipour & Fischer, 2011), and Chinese individuals, who predominantly used only right hand while counting to ten, wherein numbers 1–5 were counted beginning from an index finger, and 6–10 by using unique symbolic gestures; see Domahs et al., 2010; Morrissey et al., 2016). Regarding the techniques used, in the current study, there was little variation—almost all out of 380 participants declared starting from the closed palm and extending consecutive fingers. Around three-quarters of them used this as the only strategy. This result is also consistent with previous data except with studies on Japanese people. In comparison with Europeans, Japanese participants typically started counting from the open palm and bent consecutive fingers (Butterworth, 1999). However, the vast majority of reports and descriptions in the literature were based on single measurements of FC routines. Their reliability and validity for numerical cognition studies largely depend on whether they are indeed stable over time.

### Stability of finger counting routines

In the literature, to the best of our knowledge, there are no data on the intraindividual temporal stability of FC in adults. Previous studies have focused only on the stability of FC routines across development (Sato & Lalain, 2008; P. Räsänen & T. Koponen, 2010, unpublished data). Stability of the routines seems crucial for the justification of previous findings, especially those that constitute an empirical basis for far-reaching theories about the embodied foundations of numerical processing. Notably, Fischer (2008) suggested that SNAs are ontogenetically earlier than the acquisition of reading and FC habits are among the crucial factors that shape them. In this suggestion Fischer seems to implicitly assume that FC routines are stable over time too, because it is hard to imagine how unstable, unreliable and highly flexibly FC routines should form relatively stable SNAs (but see Cipora, Patro & Nuerk, 2018 for an overview of situated influences on SNAs).

Since previous reports did not directly address neither declared nor actual stability of FC routines, we investigated this issues in our survey. The series of questions directly or indirectly concerned participants’ declared stability of their FC routines. It appeared that the majority of participants viewed their FC technique as stable and half of them declared using their preferred hand for counting even when it is full. Moreover, those measures were congruent—the proportion of participants using the preferred hand in any circumstances was related to declared stability. Also, counting against one’s typical routine turned out to be more natural and comfortable for those declaring no problems using one’s non-preferred hand for FC. Nevertheless, all these data come from self-report, and as shown by Lucidi & Thevenot (2014), such reports might not be accurate in the case of FC. Therefore, we used an additional means for testing temporal stability, testing the same participants for the second time.

The majority of the participants’ FC behavior in our study, indeed, appeared stable when tested 2 months following the first session. Interestingly, stability was related to the starting-hand in the first measurement. Individuals who started with the right hand in the first measurement were more stable than individuals, who started with the left hand. As pointed out above, it is possible that some who reported starting from the left hand in questionnaires were in fact right starters—this could explain the divergence between the findings about FC starting habits in the current and in Wasner et al. (2014) study.

It is worth to emphasize that the procedure of our study was not identical to the visual perception condition in Wasner et al. (2014). In the Wasner et al.’s visual perception condition, the participants were instructed to hold their hands in front of them and then to count, while in the current study, participants, before getting the questionnaires, were orally instructed to have their hands free and to keep their arms down. Thus, they were not holding the hands in front of them, and they did not see them.

It is conceivable that the visual perception of fingers lined up from left-to-right—like in the visual perception condition in the study by Wasner et al. (2014)—could affect at least some participants that they are more likely to count from left-to-right. The data seem to support this interpretation: in the Wasner et al.’s visual perception condition, 54% of the participants started FC with their left hand, and in our sample, it was only 43% (chi_1_^2^ = 7.81, *p* = 0.005). However, cross-cultural differences cannot be theoretically excluded, although, there is no theoretical reason (such as a different reading/writing direction) to predict such a difference between Polish and German participants.

Our procedure also differs from the spontaneous FC condition in Wasner et al. (2014) study, in which the experimenter observed how the participants are counting. Thus, there was no left-to-right presentation of hands anywhere in this condition. In the current study, the participants did not have a visual perception of their hands in front of them while counting, but they did have such a perception when reporting how they counted on the paper. This might have influenced their reports if they did not remember well, how they counted or decided to report a different counting direction when they had the schematic presentation of their hands in front of them. The results again support this suggestion. In the Wasner et al.’s spontaneous condition 28% of participants started FC with the left hand, while in the current study 43% did so (chi_1_^2^ = 5.17, *p* = 0.023). This significant difference suggests that the schematic presentation of the hands in the report also influenced performance.

All these subtle differences raise the question, how to best assess FC in a way that the type of assessment does not influence the reports of the habits too much. In this study, we conducted a group study, because we wished to investigate large-scale numbers of participants. By our instruction, we wanted to reduce the influence of visual perception during the counting process. In the report, we used the standard way of reporting counting preferences. Given our results, it can be critically discussed whether the depiction of hands and fingers should be different to assess FC preferences in a group study. We suggest that there is no simple solution to this issue. If, for instance, one would put the hands vertically, subjects might be tempted to name the fingers from top-to-bottom, because this is the standard way of numerical lists and tables in our reading culture. Additionally, they have to perform a mental rotation, in which the left and right hand should not be mixed up. Therefore, having the hands in vertical schema might not be optimal either. Furthermore, any types of a verbal list might bias participants to report congruent to the order of the list.

The report of FC preferences is at least in some participants induced by the way it is assessed. In our view, this certainly merits further investigation. Not only the “true” FC preferences (if they exist) should be uncovered by the best possible assessment, but beyond that this raises the question about embodied cognition, that is, how the perception and sensation change our cognition and memory about usual numerical counting habits.

### Finger counting use—exploratory analysis

When it comes to FC, apart from how, it is important to determine when it is actually used and to provide quantitative estimates. In the case of our subjects, FC is used most often to list elements (e.g., counting the number of guests at the party) and to present one’s arguments or plans to others. In these two aspects more than 40% of participants declared that they use FC often or very often. FC is also quite commonly used for calendar calculations (e.g., determining what day of the week is the deadline for submitting conference abstracts) and communicating quantity. These cases correspond with typical examples mentioned in the literature (Butterworth, 1999). In our study, the least prevalent use of FC use was for multiplication—77% of participants declared to never use it mdash; which is congruent with everyday observations indicating that currently such a calculating method is used very rarely despite its wide popularity in past decades (Ifrah, 1981; Butterworth, 1999). Relatively low on the FC use list were simple arithmetical operations. These results show that the use of fingers in adults rather serves as a means to reduce working memory load and to support both cardinal and ordinal aspects of processing numerical information (e.g., enumerating objects).

Differences between various subgroups, for example, individuals who use their fingers for simple arithmetical operations vs. participants who do not do it, should be subject to further research. The observed gender difference (females declaring more often using FC and males declaring more frequent finger montring), also desires further investigation. The comparison between participants who declared to be stable vs. unstable in FC habits seems justified, since the results of our study indicate that self-report measures are indeed fairly accurate. The question of how the particular groups differ regarding various number processing-related features deserves more attention in future research. Such research should address, in our opinion, not only the mutual relationship between the stability of FC habits and SNA, but also its relation to math skills and approximate number system measures.

## Conclusion

Many publications emphasize the following claims about FC: it is widespread across the vast majority of cultures since prehistoric times (Göbel, Shaki & Fischer, 2011; Overmann, 2014); it played an important role in the development of Western mathematics (Ifrah, 1981); it is extremely important in the acquisition of numerical competences (Piaget, 1942; Gelman & Gallistel, 1986; Jordan et al., 2008); it affects adult numerical processing directly (Domahs et al., 2010; Klein et al., 2011; Previtali, Rinaldi & Girelli, 2011); and finally, it is a splendid manifestation of the embodiment of mathematics (Fischer & Brugger, 2011; Fischer, 2012), as shown, for example, by the finding that simple mental calculations are realized through off-line simulation of finger movements (Andres, Seron & Olivier, 2007).

While the benefits and influences of FC have been often and controversially discussed (Moeller et al., 2012), we do not know so much about the attributes of FC. Here, we show that default FC habits are relatively stable in the majority of participants (to the best of our knowledge for the first time in a test–retest design). Moreover, most participants report that they are, nevertheless, relatively flexible in changing their FC habits when necessary (e.g., when holding an object) and that actual finger use is still widespread in healthy adults for a variety of activities. We conclude that these findings underline the practical significance of FC throughout the life-span.

## Additional Information and Declarations

### Data Availability

The following information was supplied regarding data availability: Cipora, Krzysztof, Mateusz Hohol, Kinga Wołoszyn, and Hans-Christoph Nuerk. 2018. “Finger Counting—Temporal Stability.” OSF. September 14. DOI 10.17605/OSF.IO/RQHFK.
